# GCparagon: evaluating and correcting GC biases in cell-free DNA at the fragment level

**DOI:** 10.1093/nargab/lqad102

**Published:** 2023-11-18

**Authors:** Benjamin Spiegl, Faruk Kapidzic, Sebastian Röner, Martin Kircher, Michael R Speicher

**Affiliations:** Institute of Human Genetics, Diagnostic and Research Center for Molecular BioMedicine, Medical University of Graz, 8010 Graz, Austria; Institute of Human Genetics, Diagnostic and Research Center for Molecular BioMedicine, Medical University of Graz, 8010 Graz, Austria; Exploratory Diagnostic Sciences, Berlin Institute of Health (BIH) at Charité-Universitätsmedizin Berlin, 10117 Berlin, Germany; Exploratory Diagnostic Sciences, Berlin Institute of Health (BIH) at Charité-Universitätsmedizin Berlin, 10117 Berlin, Germany; Institute of Human Genetics, University Medical Center Schleswig-Holstein (UKSH), University of Lübeck, 23562 Lübeck, Germany; Institute of Human Genetics, Diagnostic and Research Center for Molecular BioMedicine, Medical University of Graz, 8010 Graz, Austria; BioTechMed-Graz, 8010 Graz, Austria

## Abstract

Analyses of cell-free DNA (cfDNA) are increasingly being employed for various diagnostic and research applications. Many technologies aim to increase resolution, e.g. for detecting early-stage cancer or minimal residual disease. However, these efforts may be confounded by inherent base composition biases of cfDNA, specifically the over - and underrepresentation of guanine (G) and cytosine (C) sequences. Currently, there is no universally applicable tool to correct these effects on sequencing read-level data. Here, we present GCparagon, a two-stage algorithm for computing and correcting GC biases in cfDNA samples. In the initial step, length and GC base count parameters are determined. Here, our algorithm minimizes the inclusion of known problematic genomic regions, such as low-mappability regions, in its calculations. In the second step, GCparagon computes weights counterbalancing the distortion of cfDNA attributes (correction matrix). These fragment weights are added to a binary alignment map (BAM) file as alignment tags for individual reads. The GC correction matrix or the tagged BAM file can be used for downstream analyses. Parallel computing allows for a GC bias estimation below 1 min. We demonstrate that GCparagon vastly improves the analysis of regulatory regions, which frequently show specific GC composition patterns and will contribute to standardized cfDNA applications.

## Introduction

Detailed analyses of circulating cell-free DNA (cfDNA) are increasingly used to detect, diagnose and monitor an array of pathological and physiological processes (1–5). However, all of these analyses depend on an unbiased representation of cfDNA. A known confounding factor is fragment guanine and cytosine (GC) sequence content-based overrepresentation (‘GC bias’) in the analyte, which is frequently introduced during library preparation, particularly during polymerase chain reaction (PCR) amplification and binding-based purification or enrichment steps, resulting in an underrepresentation of regions that are either extraordinarily GC-rich or GC-poor (6). This GC bias may vary considerably between samples, and variation in GC bias has even been found between libraries generated from the same starting material (7). As, for example, PCR-free approaches to library preparation are currently impractical for cfDNA due to the low amounts of input DNA, GC bias represents an inevitable problem in cfDNA analyses.

Genome segmentation-based GC bias corrections have been successfully implemented for establishing copy number alterations from cfDNA (8–10). However, these approaches are not applicable to locus-based analyses as local GC content may differ greatly from the genome-wide average distribution.

As alternative solution, a fragment-based GC bias correction was previously introduced (7) and implemented in the deepTools suite (https://deeptools.readthedocs.io/en/develop/) (11) for chromatin or transcription factor (TF) immunoprecipitation data. As such data are experimentally fragmented (e.g. physical or enzymatic treatment of genomic DNA from cells or tissues) with a narrow length distribution, the deepTools implementation considers only one representative (mean) fragment length. This disregards any length variability naturally present from the biological processes that contribute to cfDNA or that might be present in different cfDNA datasets.

A method termed Griffin adapted the Benjamini and Speed GC bias correction (7) within a computational framework for profiling nucleosome protection and accessibility from cfDNA (12). However, the Griffin algorithm excludes 18.6% of the reference genome from analysis based on the unique mappability track. Furthermore, the GC bias computation is hindered by the necessity of a separate precomputed GC frequency file specifically for the reference genome build and the included regions. Moreover, the Griffin framework requires the user to edit multiple pipeline configuration files for each batch of samples and directly aggregates GC-corrected values. It does not offer the option to modify the alignments [i.e. binary alignment map (BAM) file] containing weights in the form of read-level tags for correction, hindering integration into other analysis pipelines and applying alternative read aggregation methods.

Hence, there is a lack of stand-alone GC bias correction tools that are especially suitable to cfDNA and reliably preserve fragmentomics signals, like the accessibility of regulatory open chromatin regions or fragment length shifts across genomic regions. We aimed to fill this gap and to develop a GC correction procedure with particular features, such as correction on the fragment level based on both GC content and fragment length, minimal exclusion of genomic regions, performance, simplicity of use and seamless integration into bioinformatics pipelines.

## Materials and methods

**Figure 1.**
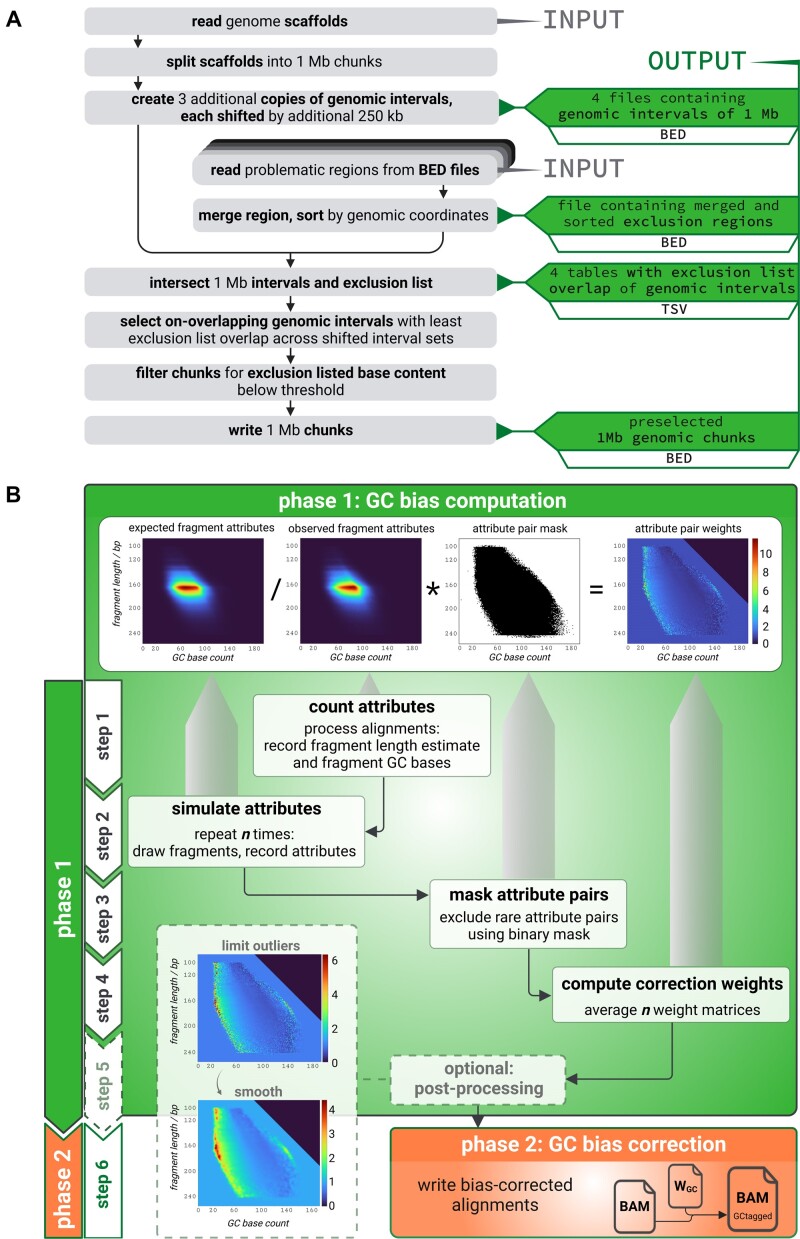
GCparagon: genomic region pre-selection and outline of algorithm. (**A**) Genomic region pre-selection: Individual BED (browser extensible data) files of exclusion masked regions (array of stacked fields next to second ‘INPUT’ mark) are combined into a single merged BED file containing non-overlapping, non-adjacent regions. Genomic scaffolds are read from a genome TSV (tab-separated values) file (left column, first ‘INPUT’ mark) and split into intervals of 1 Mb. Splitting is repeated three times, with an increasing offset of 250k bases. These four files are intersected with the exclusion marked regions, generating four tables as output. Intervals are sorted in ascending order of overlap percentage. Top bins are selected up to a specified threshold on the exclusion overlap. Genomic intervals overlapping any previously selected ones are skipped. Finally, a file of predefined 1 Mb intervals is generated, which is used by GCparagon. Figure created with BioRender (biorender.com). (**B**) The two main phases of the GC bias correction by GCparagon. In phase 1 (upper section), the GC bias is computed in the form of correction weights, which are the reciprocal of the corresponding bias values. This is done in four or five steps (big upper box including box containing heatmaps; vertical block arrow ‘phase 1’ on the left and block arrows ‘step 1’ to ‘step 5’). In the second phase (bottom section), a weight-tagged BAM file is created from the input using the computed correction weights (bottom right box; bottom left vertical block arrow ‘phase 2’ and block arrow ‘step 6’). The BAM input is processed in pre-selected genomic intervals using parallel processing in step 1 (box ‘count attributes’) and step 2 (box ‘simulate attributes’). In step 1, the fragment attribute pairs ‘observed template length’ and ‘GC base count’ are recorded in the observed attribute matrix (heatmap ‘observed fragment attributes’) for each fragment passing alignment filters from the input file. Simulated attribute pair counts (heatmap ‘expected fragment attributes’) are obtained in the second step by sampling the reference genome sequence equally using the fragment length distribution that was observed in the same genomic interval. Simulation is repeated *n* times to create *n* expected fragment attribute matrices. A binary mask (plot ‘attribute pair mask’) is created in step 3 (box ‘mask attribute pairs’) based on a minimum attribute pair count threshold applied to both the observed and expected attribute matrices. In step 4 (box ‘compute correction weights’), the binary mask is used to exclude both rare and absent attribute pairs from the computation of the *n* correction weight matrices (rightmost heatmap ‘attribute pair weights’). The latter are computed by element-wise division of the expected fragment attribute matrix by one of the *n* observed attribute matrices. The *n* correction weight matrices are consolidated by element-wise averaging across matrices. The resulting consolidated weight matrix can be post-processed in an optional fifth step (box ‘optional: post-processing’) to reduce the effect of outliers and noise on the correction (boxes with dashed edges). In the second phase, a weight-tagged version of the input BAM file is generated for downstream analysis. The (post-processed) consolidated weight matrix downstream analysis is used for tagging. Figure created with BioRender (biorender.com).

Our algorithm operates over predefined, reference genome-specific regions of equal size, which are automatically selected, and a sophisticated procedure excludes regions known to be highly problematic for short-read alignment experiments (Figure 1A). GCparagon is then based on the principle of comparing the obtained (experimental) cfDNA data with expected results from even sampling from the human reference genome sequence (Figure 1B).

### Genomic region pre-selection

The Encyclopedia of DNA Elements exclusion list version 2 was used to define problematic regions (13). The following additional regions were excluded (using file concatenation, coordinate sorting and bedtools merge): *N*-masked gaps in the primary assembly of standard chromosomes (chr1–chr22, chrX, chrY); gaps in short chromosome arms, between contigs and between scaffolds; telomeres; centromeres; gaps caused by sequencing issues related to heterochromatic DNA; alternate (ALT) contigs; tandem repeats from the tandem repeat finder software (14); and low-mappability regions from Global Alliance for Genomics & Health (15) (https://github.com/genome-in-a-bottle/genome-stratifications). The Y chromosome and regions within 1 Mb to any chromosome end were ignored during region pre-selection. A BED file of these merged, excluded regions is generated (Figure 1A).

The genome scaffold was read and split into intervals of 1 Mb. The genome split was repeated three times, each time shifting the chromosomal start position downstream by 250 kb. Each split was saved to a BED file. The resulting four BED files were intersected with excluded regions as defined above, storing the number of overlapping bases for each genomic interval in tables. Intervals with >33% (default of a user-defined value) excluded bases were removed. From the remaining intervals from the four splits, those with the smallest overlap with the exclusion list were sorted in ascending order of their overlap and selected following this sort order. Intervals overlapping any previously selected ones were ignored.

### Algorithm description

GCparagon comprises two phases (Figure 1B), the first to calculate weights for GC bias correction and the second to add these weights to the BAM file as a tag. Both algorithm stages use multiprocessing and can be carried out independently. GCparagon uses aligned, paired-end short DNA sequence reads in BAM format (SAM format specification conforming) as input. The input BAM file must be coordinate-sorted. For properly paired reads, the observed template length reported in the BAM file is used as the best available fragment length estimate; non-proper paired reads, mates with at least one extensively clipped alignment (>25% of alignment), non-primary alignments and supplementary alignments are not considered. The input BAM file must have been created with alignment software that adheres to the SAM format specification. Especially, aligners like bwa mem, which implement the observed template length metric as in the TLEN#1 definition of the SAM format specification, are supported by GCparagon (https://samtools.github.io/hts-specs/SAMv1.pdf). For each proper paired read, only the alignment with a positive observed template length is processed to avoid double counting of fragments.

GCparagon uses two features, estimated fragment length and GC base count, of each cfDNA fragment within a user-defined fragment length range to create a count matrix. GC base count is inferred from the reference sequence corresponding to each fragment. The option to freely select the fragment length range makes GCparagon applicable to virtually all cfDNA applications. For example, users can include all Illumina-sequenceable cfDNA fragments by choosing an expected fragment length range of 20–550 bp, or if only a specific range is analyzed, as in an application such as DELFI [DNA evaluation of fragments for early interception (16)], the analysis can be limited to fragments between 100 and 220 bp.

Besides fragment length range, we added further customization options for other experimental setups. To facilitate these customization options, we defined four parameter settings, which we refer to as presets 0–3 (see Table 1). Preset 0 is the default, whereas presets 1–3 offer different settings for the parameters target number of fragments processed, simulation rounds, minimum fragment attribute pair count, outlier detection, weight smoothing and smoothing strength (details are available at https://github.com/BGSpiegl/GCparagon). Different target numbers of processed fragments have an enormous impact on computation time, but only a surprisingly little effect on the quality of the GC correction (see the ‘Results and discussion’ section).

**Table 1.** GCparagon preset definitions

Bias computation				Post-processing			Performance
Preset	Target fragments processed	Simulation rounds	Minimum attribute pair count	Outlier detection	Weight smoothing	Smoothing strength	Expected computation time
0 (default)	5 000 000	6	3	Off	Off	5 (not used)	1–3 min
1	5 000 000	6	2	On	On	5	1–3 min
2	50 000 000	4	10	On	On	2	5–10 min
3	99 999 999 999	4	20	On	On	2	Depends on BAM size

To calculate the output weights for GC bias correction, GCparagon uses the DNA intervals generated, as described in Figure 1A. Python’s multiprocessing library allows for the parallel processing of *p* intervals (large box with matrices; Figure 1B). After counting fragment attributes observed in an interval of the BAM file, the bias computation step simulates the expected GC base count, assuming each location within the interval may act as fragment origin with equal probability. During this simulation, fragment sequences are repeatedly drawn for each genomic interval following the fragment length distribution observed in the same genomic interval. Two features, fragment length and GC base count, are recorded for each observed fragment passing filters and for each simulated fragment.

Drawing a sequence from the reference genome is repeated if any *N* bases are present in the simulated fragment sequence. If 55 or one-third (whichever is higher) of the sequences for any fragment length need to be redrawn, the affected genomic bin is discarded. Hence, rare fragment lengths not occurring at least 55 times in a genomic interval cannot cause a genomic interval to be dropped during simulation. The chosen number 55 represents a trade-off between allowing poorly pre-selected genomic intervals (i.e. containing many ‘*N*’ bases) to be automatically removed from the GC bias computation while not over-rejecting intervals with only a few *N*s. The optimum of this parameter generally depends on the chosen size of pre-selected genomic intervals. We empirically established the upper threshold of 55 during the testing of the algorithm. We found it appropriate for the provided pre-selected genomic intervals and whole-genome sequencing (WGS) samples. The feature combination counts obtained by random sampling are stored separately for each simulation round.

After reaching, for example, 5 million processed fragments for preset 1 or exhausting the list of genomic intervals, the resulting matrices are combined across all processed regions by adding all *NS*_GC_ matrices for a specific simulation round *n* and by adding all *NO*_GC_ matrices. This yields one *O*_GC_ matrix and *nS*_GC_ matrices, one for each simulation round.

GC bias correction weights are then computed as follows: For each simulation round *n*, the *S*_GC_ matrix is divided element-wise by the *O*_GC_ matrix. Feature combinations missing from either the *O*_GC_ or the *S*_GC_ matrix or that occurred only rarely (e.g. once for preset 1) are masked out before division. Masked attribute combinations remain uncorrected with a default weight of 1.

Weight computation is done for all *n* simulation rounds separately. The element-wise average across weight matrices is computed afterward to reduce random effects, which could otherwise manifest as outliers. Such outlier weights result, for example, from randomly drawing ultra-rare attribute combinations, which may be overrepresented in the experimental data. However, averaging does not remove extremely high weights arising from experimentally highly underrepresented attribute combinations observed regularly from random draws.

Thus, extremely high outlier weights require post-processing. The first post-processing step computes and limits extreme outliers to an upper weight threshold *W*_threshold_ based on Equation (1), where *W*_Q3_ is the third quartile of non-default weights and IQR_W_ is the interquartile range of non-default weights. The IQR stringency factor *f*_s_ can be user-defined with the outlier detection stringency command line parameter *S*_od_ [Equation (2)], where a maximal stringency value of 7 results in an *f*_s_ of 3, effectively limiting the largest amount of non-default weights to the computed threshold.


(1) \begin{eqnarray*}{{{W}}}_{{\mathrm{threshold}}} = {{{W}}}_{{\mathrm{Q3}}} + {{{f}}}_{\mathrm{s}}\times{\mathrm{IQ}}{{\mathrm{R}}}_{\mathrm{W}},\end{eqnarray*}

(2) \begin{eqnarray*}{{{f}}}_{\mathrm{s}} = 10 -{{{S}}}_{\mathrm{od}}.\end{eqnarray*}

In the second post-processing step, smoothing is applied to the weight matrix by convolution with a two-dimensional Gaussian kernel. This mitigates random effects, e.g. from stopping the bias computation early, like for preset 1 computations, and effects that might arise from alignment artifacts or fragment length estimation inaccuracies via the observed template length because of the presence of indels. Weight matrices from each post-processing step are saved to text files (correction matrix) and can be used for subsequent alignment tagging. Visualizations of the weight matrices, counts of fragment attribute combination, the weight computation mask and the observed fragment length distribution can be set to be created automatically.

A GC bias-minimized depth of coverage (DoC) signal across genomic loci can be obtained using GC tag sums instead of raw fragment counts, increasing the comparability between loci and samples of similar average DoC. Alternatively, fragment weights can be used directly from the correction matrix text file in combination with alignments from the input BAM file in any custom code, obviating the need to write a new BAM file. However, in our experience, repeatedly associating alignments with GC bias correction weights is slower than writing the tagged BAM file once and using tagged alignments instead. Further, the need to use two linked files instead of one increases the risk of user errors.

### Benchmarking and validation

The computation time of 683 samples processed on a SLURM (Simple Linux Utility for Resource Management) high-performance computing (HPC) cluster was extracted from log file time stamps. Expected fragment GC content distribution for each sample was computed by drawing 500 million fragments equally distributed over all chromosomes, excluding Y, following a sample’s observed fragment length distribution.

The total number of DNA fragments overrepresented by a distorting sample processing step, like PCR, must be identical to the number of underrepresented fragments by the same process, assuming that fragment abundance in the original population is not limiting. According to this assumption, we computed the fidelity of our GC bias correction by comparing the total fragment count in the *O*_GC_ matrix to the sum of all weights in the computed *W*_GC_ matrix. We also corrected GC bias in samples B01, H01, C01 and P01 using the Griffin algorithm and compared the results to ours (Supplementary Tables S1-S3).

### Coverage analysis of regulatory regions

DoC signals for transcription start site (TSS) and transcription factor binding site (TFBS) loci were determined as described previously (17). Unmapped reads, alignments with mapping quality zero and supplementary alignments were not considered. The 10 000 most reported TFBSs were extracted from the Gene Transcription Regulation Database (GTRD) version 21.12 (18). TSS loci were oriented left to right in 5′ → 3′ direction according to the gene’s strand. Fragment sizes between 110 and 210 bp were included for TSS and TFBS plots. Coverages were normalized for visualizations by dividing the coverage of each locus by the average coverage across two 1.5 kb regions, each starting 1.5 kb up- or downstream to the site of interest. Of these normalized coverage values, the base-wise mean across regions of the values between the 10th and the 90th percentiles was taken. An adapted version of pysam was used to directly extract GC correction weights from alignment tags to compute the fragment’s central 60 bp DoC. The adapted version is available at https://github.com/Faruk-K/pysam.

### Software environment

A conda environment file is provided to install all software dependencies. We recommend using the Mamba package manager micromamba for creating the environment (http://github.com/mamba-org/mamba). GCparagon uses the following software: Python v3.10 (www.python.org), samtools v1.16 (19), scipy 1.9 (20), pysam v0.19 (https://github.com/pysam-developers/pysam) wrapping HTSlib (21), twobitreader v3.1 with py2bit v0.3 and numpy v1.23 (22). Visualizations use plotly express v0.4 (https://pypi.org/project/plotly-express). Genomic region pre-processing uses bedtools v2.30 (23) with pybedtools (http://github.com/daler/pybedtools). A reference genome file in FASTA format can be converted to the 2-bit version using UCSC (University of California, Sanata Cruz) FaToTwoBit v377 (http://hgdownload.cse.ucsc.edu or directly downloaded from https://hgdownload.soe.ucsc.edu/goldenPath/hg38/bigZips/latest). The Python memory profiler package was used to test memory consumption (http://github.com/pythonprofilers/memory_profiler).

### Reference genome

The GRCh38 human reference genome provided as hg38 by UCSC (14) (without ALT scaffolds) was used for all presented results.

### Samples used to demonstrate GCparagon’s capabilities

GCparagon was tested on 683 cfDNA samples collected from our liquid biopsy studies. Each study was approved by the Ethics Committee of the Medical University of Graz [approval numbers: 29-272 ex16/17 (healthy individuals), 21-228 ex09/10 (prostate cancer), 21-227 ex09/10 (breast cancer) and 21-229 ex13/14 (colon cancer)] and was conducted according to the Declaration of Helsinki. Written informed consent was obtained in each case. We highlight results from four samples [healthy donor (H01) and patients with breast (B01), colon (C01) and prostate (P01) cancer] in our figures.

## Results and discussion

### Genomic coverage and region pre-selection

Careful region pre-selection is vital to make GC correction broadly applicable. GCparagon uses predefined genomic bins and excludes these only if a user-defined threshold of excluded bases is reached (Figure 1A). Our default exclusion list contains a total of 361.7 Mb, corresponding to 11.9% of the GRCh38 reference genome (excluding Y chromosomal and mitochondrial sequences); hence, 88.1% of the human reference genome (2.67 Gb) remains unmasked. To the best of our knowledge, GCparagon’s default exclusion list keeps the largest proportion of the human genome in its analyses compared to other published procedures (12,24). For example, the Griffin procedure excludes 18.6% instead of our 11.9% (12).

### Effective GC correction and validation of GCparagon

**Figure 2.**
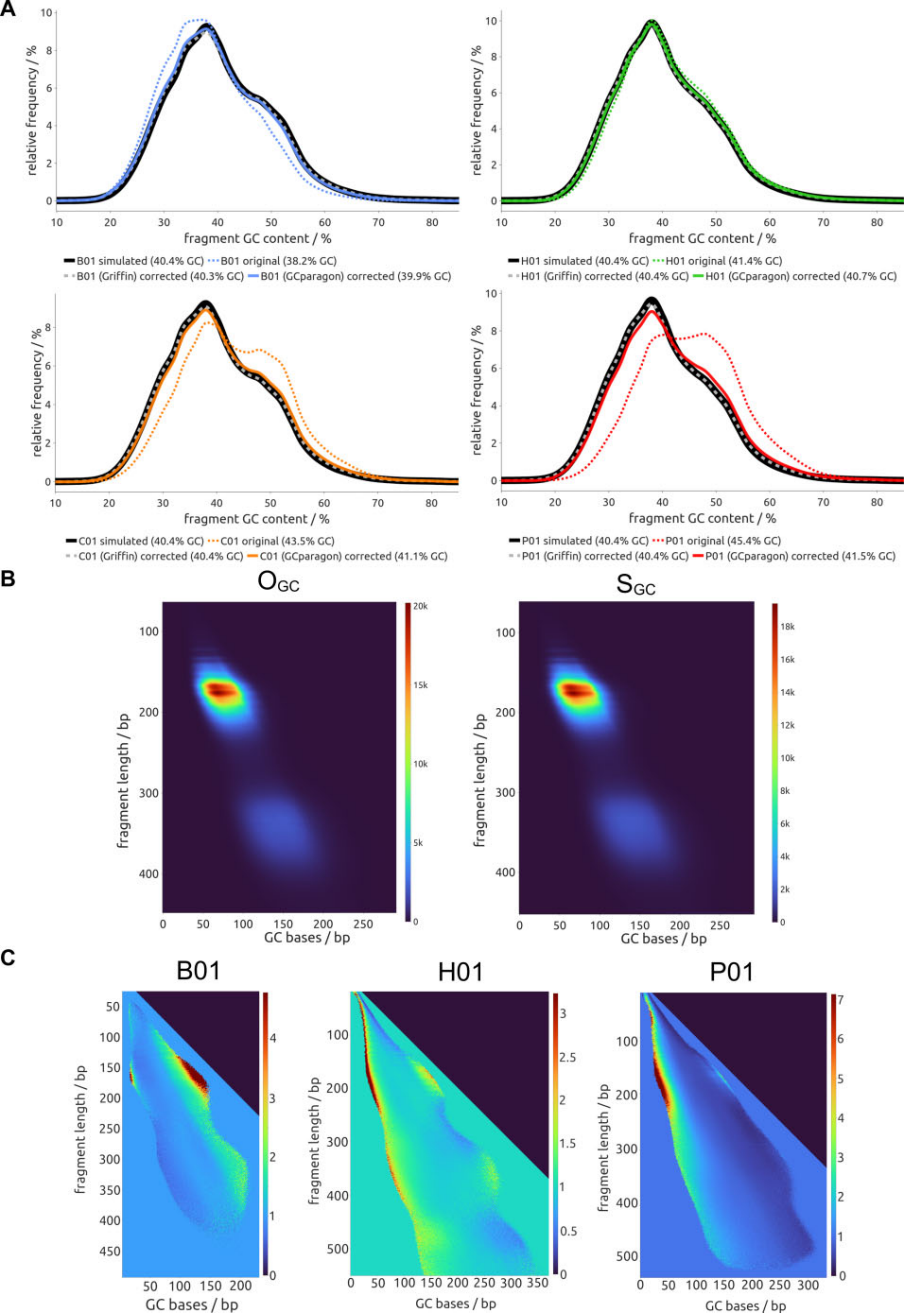
Validation of GCparagon’s GC correction performance. (**A**) Density plot of per-fragment GC content across cfDNA samples B01 (patient with breast cancer), H01 (healthy individual), C01 (colon cancer) and P01 (prostate cancer). For each sample, three lines are displayed. Dashed lines represent the original GC content for the entire input BAM file. Theoretical GC content, as established by simulating fragments across the whole genome using the observed fragment length distribution, is shown as solid, thick dark lines. GC content after correction with GCparagon is displayed as solid lines (medium thickness, lighter color), and as thick, dashed lines (medium thickness, lighter color) after correction with the Griffin algorithm. (**B**) Count matrices for observed (*O*_GC_) and randomly sampled (*S*_GC_) cfDNA fragments of a healthy individual (H01) with an uncorrected average GC content of 41.4%, computed with parameter preset 1. The *y*-axis displays the cfDNA fragment length, and the *x*-axis displays the GC base count. (**C**) Correction weight matrix of the GC correction: The *y*-axis displays the cfDNA fragment length (minimum set to 20 bp, maximum set to 550 bp). The correction value equals the reciprocal of the bias value. The visualization zooms in on non-default weights. The *x*-axis indicates the GC base count of a fragment. Impossible GC base counts receive a default value of zero and manifest as dark triangles in the upper right part of the heatmap. A color scale indicates the weights for correcting the fragment length and GC base count-specific GC bias. Red indicates a high correction weight for highly underrepresented fragments (e.g., right side of B01 and left side of P01, non-default weights in fragment length range 120 bp to 210 bp). Default weights of 1 for extremely rare fragment attribute combinations are uniformly colored (lower left part of the heatmap). The left panel displays the weight matrix of sample B01 with an average fragment GC content of 38.2%. As the sample’s GC content is below the expected 40.4% for humans, the overall GC content needs to be increased through correction, which is visible as turquoise to red regions for high GC content fragments (right part of non-default weights) and darker blue regions in the weight matrix for low GC content fragments (left part of non-default weights). The middle panel shows the H01 sample, which has a low GC bias of approximately +1%. Accordingly, the colors in this correction matrix indicate values that are closer to 1 and the upper end of the color scale is the lowest for the shown samples. The right panel illustrates the correction weights of P01 with an average GC content of 45.4%. In this case, the GC content needs to be decreased, which is visible as the inverse color pattern compared to B01. Weights were computed using preset 3.

We applied GCparagon to several hundred cfDNA samples (*n*= 683) for benchmarking and used four exemplary cfDNA samples (H01, B01, P01, C01) with various GC biases to illustrate the validity of our results (Figure 2A and B). As outlined above, we first analyze the observed GC content (*O*_GC_) of a sample and then compute the theoretical GC content employing random sampling of fragments with the same length distribution from the reference genome (*S*_GC_). GCparagon is used to rebalance the distorted GC content, and the successful GC correction should be reflected in a high similarity of the corrected fragment GC content distribution to the genome-wide simulated fragment GC content distribution, comparable to Griffin algorithm results (Figure 2A).

As expected, the impact of the GCparagon algorithm on sequencing data depends greatly on the present GC bias. For example, the average GC content of fragment sequences of the healthy control sample H01 was 41.4%, only 1.1% above the randomly sampled GC content of 40.4% (Figure 2A). Accordingly, the algorithm’s output visually confirmed the high agreement between *O*_GC_ and *S*_GC_ values (Figure 2B).

In contrast, cfDNA sample B01 had a below-human average GC content of 38.2%, reflected in a left shift of the observed fragment distribution toward lower GC contents, resulting from an overrepresentation of low-GC fragments and underrepresentation of high-GC fragments, respectively (Figure 2A). In such a case, the correction must rebalance the misrepresented fragments in a way that leads to an increased overall GC content, which GCparagon reflects and visualizes in its correction matrix (Figure 2C). The corrected GC content profile overlaps closely with the expected profile (Figure 2A). Conversely, cfDNA sample P01 had an above-human average GC content of 45.4%, visible as a shift to the right in the original fragment GC content plot (Figure 2A). In such a case, GCparagon decreases the overall GC content (Figure 2C).

Comparing the P01 correction weight matrices from GCparagon and Griffin revealed a pronounced artifact for Griffin caused by extensively soft-clipped read alignments in the low GC content range (Supplementary Figure S3).

Furthermore, we extensively tested presets 1–3 and deemed them appropriate for WGS samples with a DoC between 0.5× and 60×. Comparing the total fragment count in the *O*_GC_ matrix to the sum of all corrected weights for the four samples results in an average deviation of +0.58%, −0.04% and −0.09% for presets 1, 2 and 3, respectively. In contrast, Griffin fragment pool size changes were much more extensive (reduction up to 45% for B01; see Supplementary Table S3).

Overall, GCparagon achieved a close approximation between corrected and simulated GC content in all tested cases.

### Potential use of the weight matrix

A GC bias-minimized DoC signal across genomic loci can be obtained using GC tags of fragments instead of raw fragment counts, increasing the comparability between loci and samples of similar average DoC. Alternatively, fragment weights can be used directly from the correction matrix text file in combination with alignments from the input BAM file in any custom code, obviating the need to write a new BAM file. However, in our experience, repeatedly associating alignments with GC bias correction weights is slower than writing the tagged BAM file once and using tagged alignments for further analysis. Moreover, using two linked files instead of one standard input file increases the risk of user errors.

### Time complexity and memory footprint

The GC bias computation using preset 1 with a target fragment number of 5 × 10^6^ is very fast. All tested 683 samples finished in under 3 min on an 11-node SLURM HPC cluster, with 58.3% of samples taking 70 s or less (Supplementary Figure S1A). Computation time is linear in the number of processed fragments (Supplementary Figure S1B). Memory consumption is linear in the number of cores used for parallel processing and the number of sampling rounds per genomic interval. Memory usage was around 2.5 GiB using 12 cores (∼200 MB per process) and independent of the number of processed fragments (Supplementary Figure S2A). Maximum consumption of 3.8 GiB was observed for P01 at preset 2, with highest memory consumption memory during BAM tagging (Supplementary Figure S2B). Compared to Griffin, GCparagon correction was 62–144 times faster (endpoint: correction table output) and 19–31 times if the output of the tagged BAM file was used for comparison (see Supplementary Table S1). The memory footprint was up to 2.4 times higher for GCparagon than for Griffin but was still below 4 GiB (see Supplementary Table S2).

### GC bias and regulatory regions

**Figure 3.**
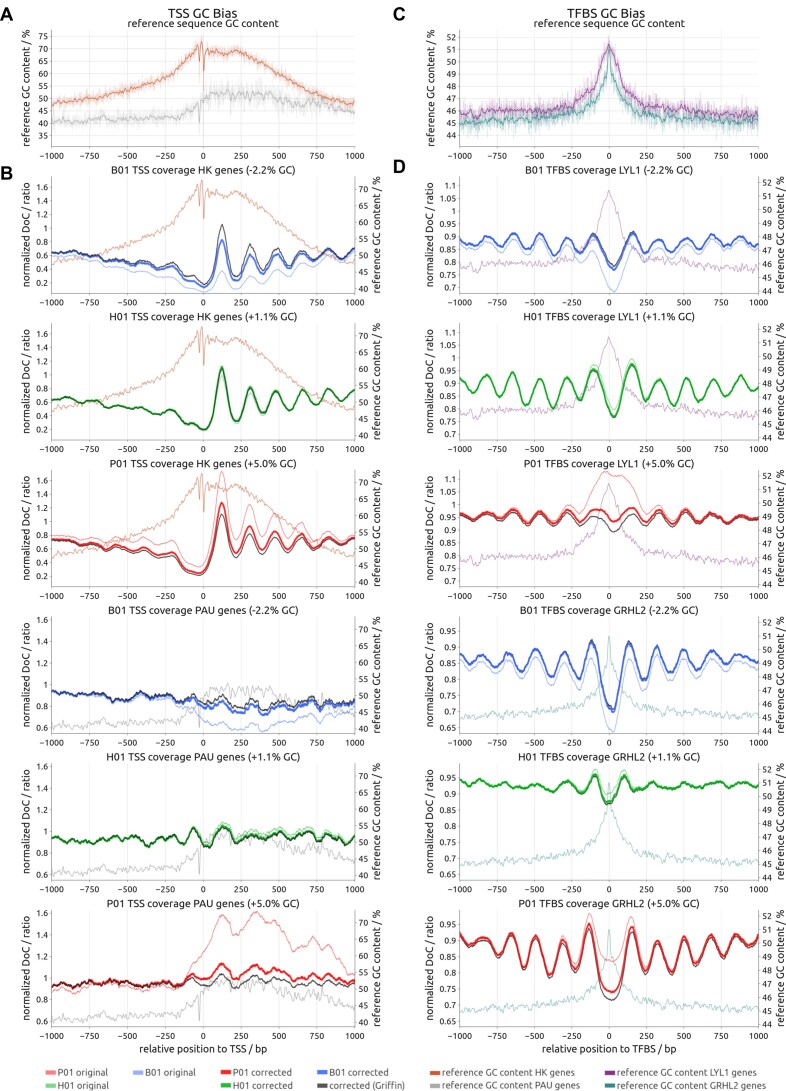
The effect of GC bias on coverage signals of regulatory regions. (**A**) GC content of the GRCh38 reference genome averaged across TSSs of HK genes (orange line; top) and PAU genes (gray line; bottom). GC content is shown twice for each gene group: unfiltered (transparent) and smoothed using a 15 bp Hamming window (opaque). A sharp GC decline upstream of the TSS (promoter region) indicates the location of the TATA box. (**B**) Original and corrected TSS coverage plots for HK genes (top three panels) and PAU genes (bottom three panels), each for samples B01 (cfDNA from a patient with breast cancer), H01 (healthy control) and P01 (prostate cancer). Coverage is computed using the fragment’s central 61 bp or fewer if the fragment is shorter. The reference genomes’ GC content plots for HK genes and PAU genes from panel (A) are included for comparison. The original coverage is shown with a thin, colored line, and the corrected coverage with a bold, colored line. The coverage after correction with the Griffin algorithm is shown as solid black line. (**C**) GC content of the GRCh38 reference genome averaged across the top 10 000 TF binding sites reported in the GTRD of two GRHL2 (green line; bottom) and LYL1 (purple line; top), respectively. GC content is shown twice for each TF: unfiltered (transparent) and smoothed using a 15 bp Hamming window (opaque). Both GRHL2 and LYL1 TFBSs show highly increased central GC content. (**D**) Original and corrected TFBS coverage plots (coverage computed using central 60 bp of fragments if 60 bp or larger) for LYL1 (top three samples) and GRHL2 (bottom three panels) from the same cfDNA samples as in panel (B). The genome reference GC content plots for the TFBSs from panel (C) were included for comparison. The coverage after correction with the Griffin algorithm is shown as solid black line.

Overlays of multiple regulatory open chromatin regions, such as TSSs and TFBSs, have been reported to have non-uniform GC content between the binding site and flanking regions (25) due to shared sequence motifs and other shared sequence features of the stacked regions. Therefore, we examined the GC content of the human reference genome at TSSs (Figure 3A) and TFBSs (Figure 3C). We used the TSSs of 1228 housekeeping (HK) genes (26) and 1172 genes that are unexpressed according to the Protein Atlas (www.proteinatlas.org) [Protein Atlas unexpressed (PAU) genes] (27). We observed that the GC content started to increase already 250 bp upstream to the TSS of PAU genes and even further upstream for HK genes (Figure 3A). This pattern of GC content increase for exon 1 is more pronounced for HK genes than for PAU genes. Downstream to the TSS, the GC content decreases after 250 bases for HK genes, while the GC content of PAU genes stays at a moderately increased level until ∼750 bases (Figure 3A). Generally, the GC content of TSS sequences for HK genes is much higher than that of PAU genes, in line with their well-described association with CpG (5′-C–phosphate–G-3′) islands. Further, when we analyzed the TFBSs of TF GRHL2, a pioneer TF for epithelial cells (28), and the hematopoietic TF LYL1 (29), we observed that both TFBSs contain GC-rich binding motifs, which result in an increased GC content close to their binding sites (Figure 3C). Hence, non-uniform GC content at regulatory regions is an inherent characteristic of cfDNA analyses, which will cause signal distortions when combined with GC biases in the analyzed samples.

In cfDNA analyses, TSS regions of HK genes have a characteristic coverage pattern with low relative coverage at the TSS position and oscillating patterns arising from phased nucleosomes mostly downstream of the TSS (Figure 3B). In contrast, TSSs of the repressed PAU genes show less variable coverage patterns, reflecting the denser nucleosome packaging and the resulting stronger cfDNA protection (17,30). For samples without appreciable GC bias, such as H01, applying GCparagon has only a minor effect on the coverage profile (Figure 3B). However, cfDNA samples with positive GC biases display increased coverages downstream of the TSS, particularly in exon 1 regions (P01 in Figure 3B). A reverse pattern, i.e. decreased coverage, can be observed in sample B01 with negative GC bias (Figure 3B). For PAU genes, a similar but much lower distortion downstream of the TSS is observed, following the reference GC content. Again, these distortions are effectively corrected by GCparagon (Figure 3B).

Accessible TFBSs show decreased coverage at their center flanked by oscillating patterns in cfDNA analyses, indicating phased positioning of nucleosomes around the TFBS (31). However, increased GC sequence content close to the binding sites (Figure 3C) results in increased coverage between 250 bp upstream and downstream with positive GC bias (P01 in Figure 3D) or decreased coverage in samples with negative GC bias (B01 in Figure 3D). After GC bias correction, the profiles show a regular oscillating pattern (Figure 3D).

### GCparagon enables downstream analyses

Assessment of TSS and TFBS coverage plots is evolving into a new tool to draw conclusions about the accessibility of TFs from cfDNA and hence infer essential biological information from the respective samples (12,31,32). Interestingly, we found that genomic features exhibiting a weak accessibility signal, like TSSs of inactive genes or moderately bound TFBSs, were particularly prone to severe coverage distortions interfering with their effective detection and impacting their detection limits. Such distortions may even lead to false positive signals in regions of otherwise nominal coverage, as was observed for LYL1 TFBSs of P01 (Figure 3D). However, stronger accessibility signals may also show substantial distortions in some cases. Examples of such signals, which correspond to deep relative coverage drops, are the difference between the central dip and downstream peak for B01 HK genes (Figure 3B) and the difference between the LYL1/GRHL2 central dip and surrounding coverage peaks for B01 and P01, respectively (Figure 3D).

In conclusion, distortions seen for various loci generally depend on the sample’s overall GC bias, the loci’s average GC content, the specific sequence composition of overlaid loci and the locally observed fragment lengths. Our results demonstrate the importance of GC bias corrections for such applications.

## Supplementary Material

lqad102_Supplemental_File

## Data Availability

The data used in this article are available in the European Genome-Phenome Archive (EGA) at https://ega-archive.org and can be accessed with EGAS00001006963. The GCparagon code is available on GitHub at https://github.com/BGSpiegl/GCparagon. The code, including the processing of the EGA samples, is also available as a release archive in Zenodo at 10.5281/zenodo.8353753. Due to their large file size, extracted read coverage overlays before and after GC correction were deposited separately on Zenodo at https://doi.org/10.5281/zenodo.7886030.

## References

[1] Hasenleithner S.O., Speicher M.R. A clinician’s handbook for using ctDNA throughout the patient journey. Mol. Cancer. 2022; 21:81. 35307037 10.1186/s12943-022-01551-7 PMC8935823

[2] Heitzer E., Haque I.S., Roberts C.E.S., Speicher M.R. Current and future perspectives of liquid biopsies in genomics-driven oncology. Nat. Rev. Genet. 2019; 20:71–88. 30410101 10.1038/s41576-018-0071-5

[3] Ignatiadis M., Sledge G.W., Jeffrey S.S. Liquid biopsy enters the clinic—implementation issues and future challenges. Nat. Rev. Clin. Oncol. 2021; 18:297–312. 33473219 10.1038/s41571-020-00457-x

[4] Lo Y.M.D., Han D.S.C., Jiang P., Chiu R.W.K. Epigenetics, fragmentomics, and topology of cell-free DNA in liquid biopsies. Science. 2021; 372:eaaw3616. 33833097 10.1126/science.aaw3616

[5] Wan J.C.M., Mughal T.I., Razavi P., Dawson S.J., Moss E.L., Govindan R., Tan I.B., Yap Y.S., Robinson W.A., Morris C.D. et al. Liquid biopsies for residual disease and recurrence. Med. 2021; 2:1292–1313. 35590147 10.1016/j.medj.2021.11.001

[6] Aird D., Ross M.G., Chen W.S., Danielsson M., Fennell T., Russ C., Jaffe D.B., Nusbaum C., Gnirke A. Analyzing and minimizing PCR amplification bias in Illumina sequencing libraries. Genome Biol. 2011; 12:R18. 21338519 10.1186/gb-2011-12-2-r18 PMC3188800

[7] Benjamini Y., Speed T.P. Summarizing and correcting the GC content bias in high-throughput sequencing. Nucleic Acids Res. 2012; 40:e72. 22323520 10.1093/nar/gks001 PMC3378858

[8] Adalsteinsson V.A., Ha G., Freeman S.S., Choudhury A.D., Stover D.G., Parsons H.A., Gydush G., Reed S.C., Rotem D., Rhoades J. et al. Scalable whole-exome sequencing of cell-free DNA reveals high concordance with metastatic tumors. Nat. Commun. 2017; 8:1324. 29109393 10.1038/s41467-017-00965-y PMC5673918

[9] Alkan C., Kidd J.M., Marques-Bonet T., Aksay G., Antonacci F., Hormozdiari F., Kitzman J.O., Baker C., Malig M., Mutlu O. et al. Personalized copy number and segmental duplication maps using next-generation sequencing. Nat. Genet. 2009; 41:1061–1067. 19718026 10.1038/ng.437 PMC2875196

[10] Heitzer E., Ulz P., Belic J., Gutschi S., Quehenberger F., Fischereder K., Benezeder T., Auer M., Pischler C., Mannweiler S. et al. Tumor-associated copy number changes in the circulation of patients with prostate cancer identified through whole-genome sequencing. Genome Med. 2013; 5:30. 23561577 10.1186/gm434 PMC3707016

[11] Ramirez F., Ryan D.P., Gruning B., Bhardwaj V., Kilpert F., Richter A.S., Heyne S., Dundar F., Manke T. deepTools2: a next generation web server for deep-sequencing data analysis. Nucleic Acids Res. 2016; 44:W160–W165. 27079975 10.1093/nar/gkw257 PMC4987876

[12] Doebley A.L., Ko M., Liao H., Cruikshank A.E., Santos K., Kikawa C., Hiatt J.B., Patton R.D., De Sarkar N., Collier K.A. et al. A framework for clinical cancer subtyping from nucleosome profiling of cell-free DNA. Nat. Commun. 2022; 13:7475. 36463275 10.1038/s41467-022-35076-w PMC9719521

[13] Amemiya H.M., Kundaje A., Boyle A.P. The ENCODE blacklist: identification of problematic regions of the genome. Sci. Rep. 2019; 9:9354. 31249361 10.1038/s41598-019-45839-z PMC6597582

[14] Nassar L.R., Barber G.P., Benet-Pages A., Casper J., Clawson H., Diekhans M., Fischer C., Gonzalez J.N., Hinrichs A.S., Lee B.T. et al. The UCSC Genome Browser database: 2023 update. Nucleic Acids Res. 2023; 51:D1188–D1195. 36420891 10.1093/nar/gkac1072 PMC9825520

[15] Krusche P., Trigg L., Boutros P.C., Mason C.E., De La Vega F.M., Moore B.L., Gonzalez-Porta M., Eberle M.A., Tezak Z., Lababidi S et al. Best practices for benchmarking germline small-variant calls in human genomes. Nat. Biotechnol. 2019; 37:555–560. 30858580 10.1038/s41587-019-0054-x PMC6699627

[16] Cristiano S., Leal A., Phallen J., Fiksel J., Adleff V., Bruhm D.C., Jensen S.O., Medina J.E., Hruban C., White J.R. et al. Genome-wide cell-free DNA fragmentation in patients with cancer. Nature. 2019; 570:385–389. 31142840 10.1038/s41586-019-1272-6 PMC6774252

[17] Ulz P., Thallinger G.G., Auer M., Graf R., Kashofer K., Jahn S.W., Abete L., Pristauz G., Petru E., Geigl J.B. et al. Inferring expressed genes by whole-genome sequencing of plasma DNA. Nat. Genet. 2016; 48:1273–1278. 27571261 10.1038/ng.3648

[18] Yevshin I., Sharipov R., Kolmykov S., Kondrakhin Y., Kolpakov F. GTRD: a database on gene transcription regulation—2019 update. Nucleic Acids Res. 2019; 47:D100–D105. 30445619 10.1093/nar/gky1128 PMC6323985

[19] Danecek P., Bonfield J.K., Liddle J., Marshall J., Ohan V., Pollard M.O., Whitwham A., Keane T., McCarthy S.A., Davies R.M. et al. Twelve years of SAMtools and BCFtools. GigaScience. 2021; 10:giab008. 33590861 10.1093/gigascience/giab008 PMC7931819

[20] Virtanen P., Gommers R., Oliphant T.E., Haberland M., Reddy T., Cournapeau D., Burovski E., Peterson P., Weckesser W., Bright J. et al. SciPy 1.0: fundamental algorithms for scientific computing in Python. Nat. Methods. 2020; 17:261–272. 32015543 10.1038/s41592-019-0686-2 PMC7056644

[21] Li H., Handsaker B., Wysoker A., Fennell T., Ruan J., Homer N., Marth G., Abecasis G., Durbin R. 1000 Genome Project Data Processing Subgroup The Sequence Alignment/Map format and SAMtools. Bioinformatics. 2009; 25:2078–2079. 19505943 10.1093/bioinformatics/btp352 PMC2723002

[22] Harris C.R., Millman K.J., van der Walt S.J., Gommers R., Virtanen P., Cournapeau D., Wieser E., Taylor J., Berg S., Smith N.J. et al. Array programming with NumPy. Nature. 2020; 585:357–362. 32939066 10.1038/s41586-020-2649-2 PMC7759461

[23] Quinlan A.R., Hall I.M. BEDTools: a flexible suite of utilities for comparing genomic features. Bioinformatics. 2010; 26:841–842. 20110278 10.1093/bioinformatics/btq033 PMC2832824

[24] Peneder P., Stutz A.M., Surdez D., Krumbholz M., Semper S., Chicard M., Sheffield N.C., Pierron G., Lapouble E., Totzl M. et al. Multimodal analysis of cell-free DNA whole-genome sequencing for pediatric cancers with low mutational burden. Nat. Commun. 2021; 12:3230. 34050156 10.1038/s41467-021-23445-w PMC8163828

[25] Valouev A., Johnson S.M., Boyd S.D., Smith C.L., Fire A.Z., Sidow A. Determinants of nucleosome organization in primary human cells. Nature. 2011; 474:516–520. 21602827 10.1038/nature10002 PMC3212987

[26] Hounkpe B.W., Chenou F., de Lima F., De Paula E.V. HRT Atlas v1.0 database: redefining human and mouse housekeeping genes and candidate reference transcripts by mining massive RNA-seq datasets. Nucleic Acids Res. 2021; 49:D947–D955. 32663312 10.1093/nar/gkaa609 PMC7778946

[27] Uhlén M., Fagerberg L., Hallstrom B.M., Lindskog C., Oksvold P., Mardinoglu A., Sivertsson A., Kampf C., Sjostedt E., Asplund A. et al. Proteomics. Tissue-based map of the human proteome. Science. 2015; 347:1260419. 25613900 10.1126/science.1260419

[28] Jacobs J., Atkins M., Davie K., Imrichova H., Romanelli L., Christiaens V., Hulselmans G., Potier D., Wouters J., Taskiran I.I. et al. The transcription factor Grainy head primes epithelial enhancers for spatiotemporal activation by displacing nucleosomes. Nat. Genet. 2018; 50:1011–1020. 29867222 10.1038/s41588-018-0140-x PMC6031307

[29] Zohren F., Souroullas G.P., Luo M., Gerdemann U., Imperato M.R., Wilson N.K., Gottgens B., Lukov G.L., Goodell M.A. The transcription factor Lyl-1 regulates lymphoid specification and the maintenance of early T lineage progenitors. Nat. Immunol. 2012; 13:761–769. 22772404 10.1038/ni.2365 PMC3411897

[30] Snyder M.W., Kircher M., Hill A.J., Daza R.M., Shendure J. Cell-free DNA comprises an *in vivo* nucleosome footprint that informs its tissues-of-origin. Cell. 2016; 164:57–68. 26771485 10.1016/j.cell.2015.11.050 PMC4715266

[31] Ulz P., Perakis S., Zhou Q., Moser T., Belic J., Lazzeri I., Wolfler A., Zebisch A., Gerger A., Pristauz G. et al. Inference of transcription factor binding from cell-free DNA enables tumor subtype prediction and early detection. Nat. Commun. 2019; 10:4666. 31604930 10.1038/s41467-019-12714-4 PMC6789008

[32] Herberts C., Annala M., Sipola J., Ng S.W.S., Chen X.E., Nurminen A., Korhonen O.V., Munzur A.D., Beja K., Schonlau E. et al. Deep whole-genome ctDNA chronology of treatment-resistant prostate cancer. Nature. 2022; 608:199–208. 35859180 10.1038/s41586-022-04975-9

