# Supplementary material for: GCparagon: evaluating and correcting GC biases in cell-free DNA at the fragment level
Source: NAR Genom Bioinform. 2023 Nov 18;5(4):lqad102. doi: 10.1093/nargab/lqad102 (PMC10657415; doi:10.1093/nargab/lqad102)
Supplement: lqad102_Supplemental_File [file lqad102_supplemental_file.pdf]

GCparagon: Evaluating and correcting GC biases in cell-free DNA at the fragment level

Supplementary Figures and Tables

Supplementary Figure 1

Time complexity of GCparagon

(a) Computation times using GCparagon with preset 1 on 683 WGS cfDNA samples. Time was extracted from log file timestamps, and computation was carried out on an 11-node HPC system. Variations in processing time can arise due to extensive node and/or network storage usage by other processes.

(b) GCparagon first stage computation time relative to the number of processed fragments. All four samples were processed using three parameter presets and evaluated using a profiling script. Bias estimation uses an increasingly larger portion of the input dataset by increasing preset number (right panel). Preset 3 uses all predefined genomic regions with an exclusion list overlap below 33%. This leads to the inclusion of a different number of fragments based on the DoC of a sample and, therefore, differences in computation time. Results were computed twice, and a linear time dependency on the number of processed fragments observed (left panel).

Supplementary Figure 2

Memory footprint of GCparagon

(a) Memory usage across samples and presets by all processes during the first stage of the algorithm using 12 cores. Memory consumption does not depend on the number of processed fragments.

(b) Memory usage over time during both stages of the GCparagon algorithm using preset 2 settings and 12 cores. Memory usage stays approximately constant during each stage with the BAM tagging stage consuming more memory. Memory consumption peaks at the beginning of the second stage when parallel processes are spawned.

Supplementary Figure 3

Softclipped alignments artifact

Comparison between correction matrices created with GCParagon, preset 2 (left) and the Griffin algorithm (right) for the highly biased sample P01. An unexpected patch of correction values is present int the Griffin correction matrix (red arrow). GCparagon ignores alignments with a softclipped fraction above 25%. The artifact is absent in the GCparagon matrix (blue arrow).

Supplementary Table 1

Griffin benchmark: computation time of GC correction algorithms

The duration of GC bias correction was reported for Griffin using the snakemake benchmark directive. The duration of correction for the GCparagon algorithm was reported by the memory_profiler software. The average across 3 repetitions is shown for each sample. Both algorithms used 12 cores. GCparagon was run with preset 2 settings. The ratio of computation time of Griffin over GCparagon is listed. Maximum value of each column is highlighted.

Supplementary Table 2

Griffin benchmark: RSS memory footprint of GC correction algorithms

Memory consumed by the Griffin algorithm, GCparagon algorithm during phase 1 only and also across both phases (GC bais computation and BAM tagging) were recorded using 12 cores and preset 2 settings for GCparagon. The average of the maximum observed memory consumption across 3 repetitions of GC bias correction is listed in MiB. The ratio of memory consumption of GCparagon over Griffin is listed. Maximum value of each column is highlighted.

Supplementary Table 3

Griffin benchmark: fragment count difference after correction

The number of observed fragments in a dataset was compared to the sum over the result of *correction matrix * observed count matrix* after GC bias correction with either Griffin algorithm or GCparagon. Fragment count differences are presented as percentages. Maximum deviations are highlighted.

Supplementary Figure 1

a

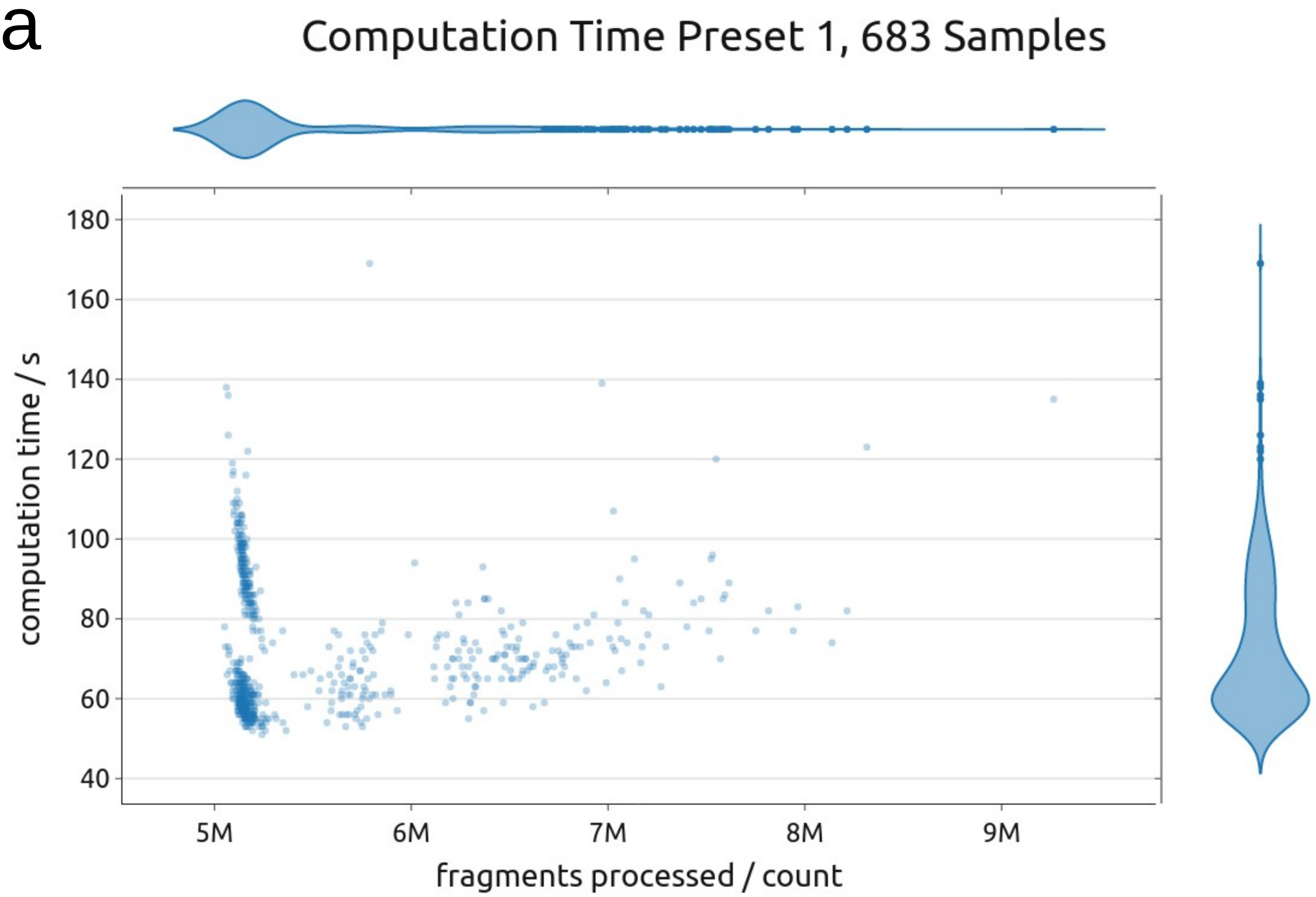

b

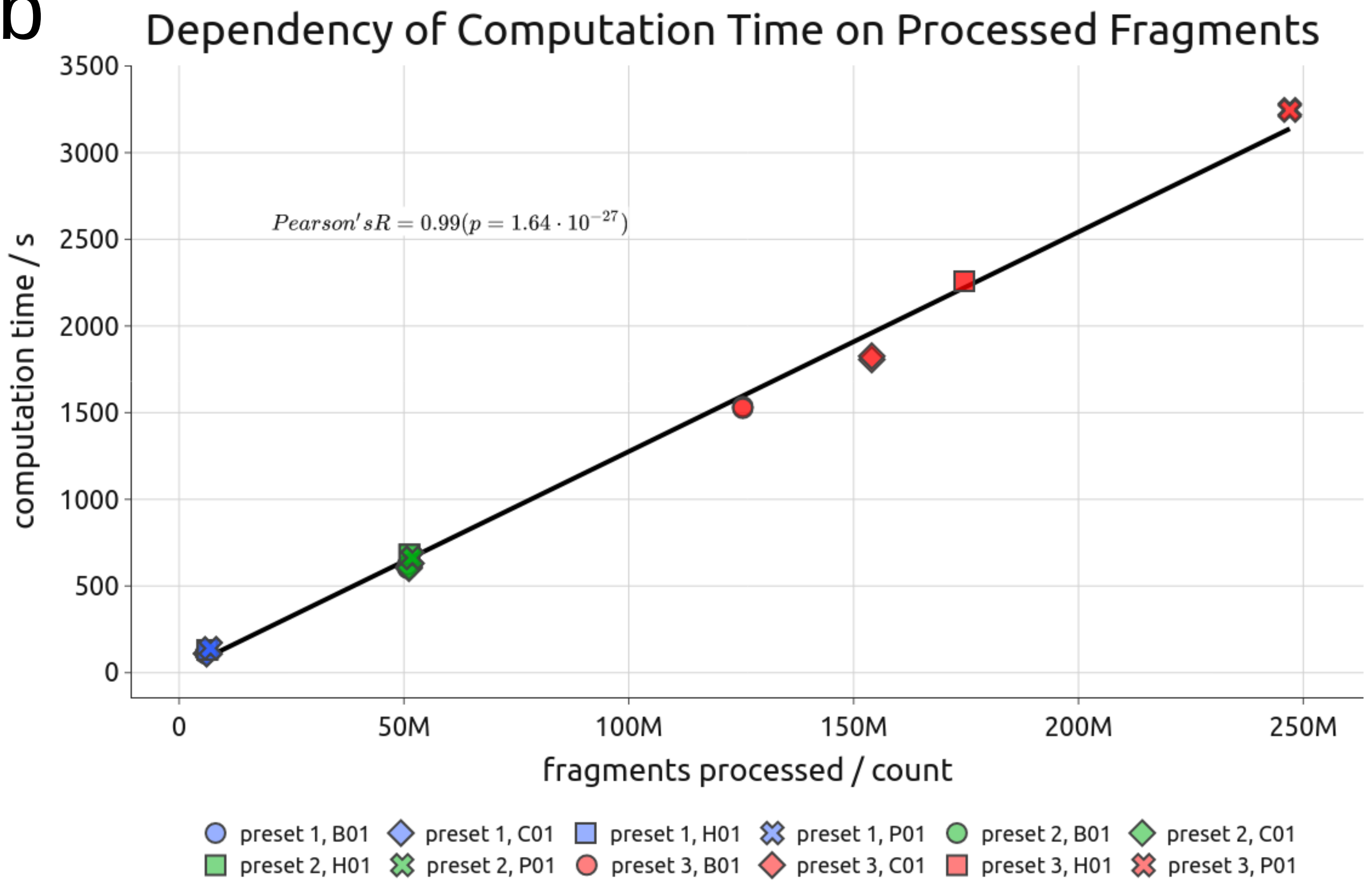

Supplementary Figure 2

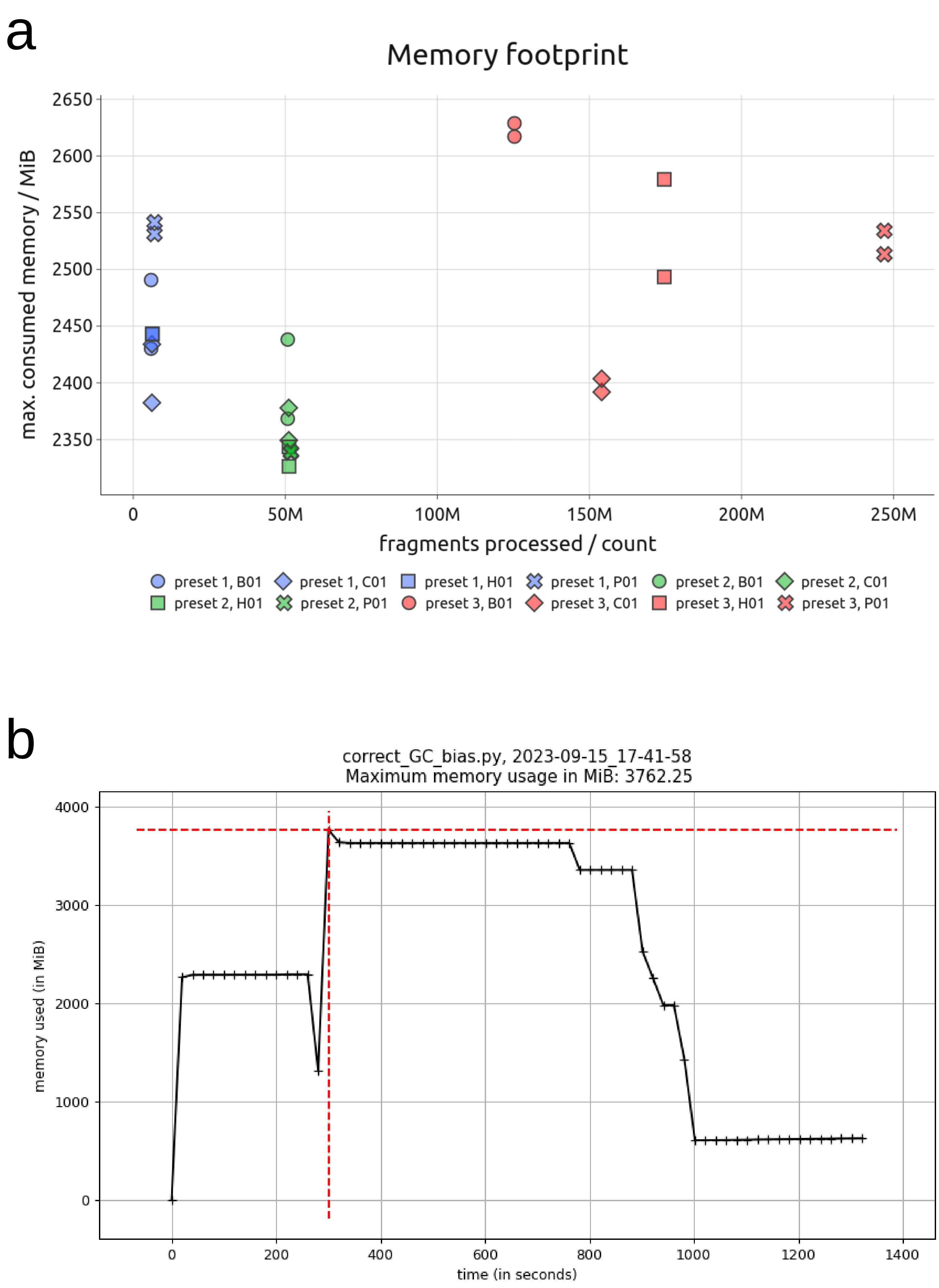

Supplementary Figure 3

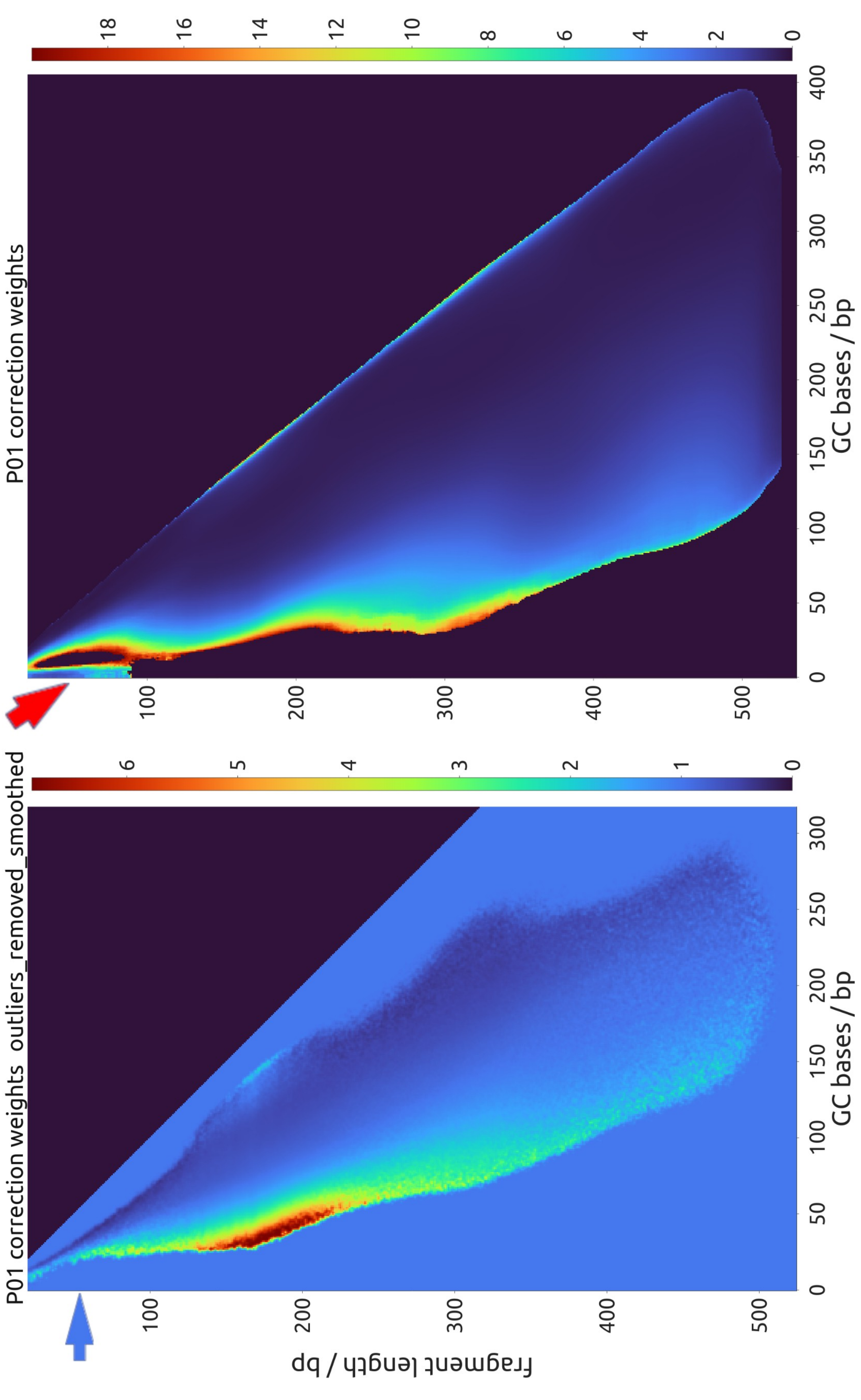

Supplementary Table 1

**Sample**	**Griffin** hh:mm:ss	**GCparagon** hh:mm:ss	**Griffin / GCparagon**	**GCparagon w. BAM** hh:mm:ss	**Griffin / Gcparagon w. BAM**
B01	04:51:39	00:04:43	61.8	00:15:32	18.8
H01	06:51:43	**00:05:14**	78.7	00:20:09	20.4
C01	05:13:52	00:04:32	69.2	00:17:09	18.3
P01	**11:54:20**	00:04:58	**143.8**	**00:23:03**	**31.0**

Supplementary Table 2

**Sample**	**Griffin** MiB	**GCparagon** MiB	**GCparagon / Griffin**	**GCparagon w. BAM** MiB	**GCparagon w. BAM / Griffin**
B01	1555	**2325**	**1.50**	3704	2.38
H01	1550	2287	1.48	3756	**2.42**
C01	1552	2272	1.46	3652	2.35
P01	**1608**	2309	1.44	**3761**	2.34

Supplementary Table 3

**sample**	**Griffin** %	**GCparagon (preset2)** %
B01	**-44.88**	**-0.11**
H01	-35.57	-0.05
C01	-24.70	-0.04
P01	-12.68	+0.04
